# A Secure, Energy- and SLA-Efficient (SESE) E-Healthcare Framework for Quickest Data Transmission Using Cyber-Physical System

**DOI:** 10.3390/s19092119

**Published:** 2019-05-07

**Authors:** Ashutosh Sharma, Geetanjali Rathee, Rajiv Kumar, Hemraj Saini, Vijayakumar Varadarajan, Yunyoung Nam, Naveen Chilamkurti

**Affiliations:** 1 Department of Electronics and Communication, Jaypee University of Information Technology, Solan 173234, India; sharmaashutosh1326@gmail.com (A.S.); rjv.ece@gmail.com (R.K.); 2 Department of Computer Science and Engineering, Jaypee University of Information Technology, Solan 173234, India; geetanjali.rathee123@gmail.com (G.R.); hemraj1977@yahoo.co.in (H.S.); 3 School of Computing Science and Engineering, Vellore Institute of Technology, Chennai 600127, India; vijayakumar.v@vit.ac.in; 4 Department of Computer Science and Engineering, Soonchunhyang University, Asan 31538, Korea; 5 Department of CS&IT, La Trobe University, Melbourne 3086, Australia; n.chilamkurti@latrobe.edu.au

**Keywords:** quickest data transmission services, critical-healthcare services, security, green energy, service level agreement, cyber physical system, secure CPS

## Abstract

Due to advances in technology, research in healthcare using a cyber-physical system (CPS) opens innovative dimensions of services. In this paper, the authors propose an energy- and service-level agreement (SLA)-efficient cyber physical system for E-healthcare during data transmission services. Furthermore, the proposed phenomenon will be enhanced to ensure the security by detecting and eliminating the malicious devices/nodes involved during the communication process through advances in the ad hoc on-demand distance vector (AODV) protocol. The proposed framework addresses the two security threats, such as grey and black holes, that severely affect network services. Furthermore, the proposed framework used to find the different network metrics such as average qualifying service set (QSS) paths, mean hop and energy efficiency of the quickest path. The framework is simulated by calculating the above metrics in mutual cases i.e., without the contribution of malevolent nodes and with the contribution of malevolent nodes over service time, hop count and energy constraints. Further, variation of SLA and energy shows their expediency in the selection of efficient network metrics.

## 1. Introduction

**Figure 1:**
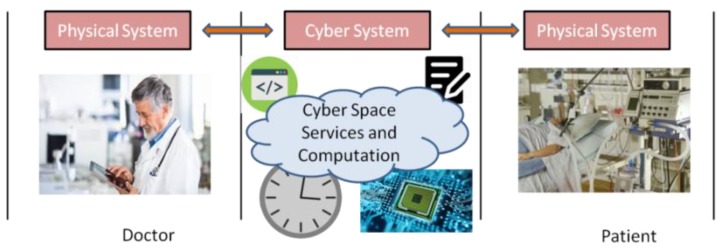
Cyber physical system (CPS) for E-healthcare systems.

In recent innovations and applications, the involvement of a cyber-physical system (CPS) can be seen widely in different areas of research such as business, intelligent driving system, tele-operated surgeries and healthcare systems. The roots of CPS is older, however and gained attention when Helen Gill through this concept in the air at the National Science Foundation (NSF). The CPS was initially introduced by Lee [1] in a NSF workshop where they discussed how physical process and computations were integrated. Presently, it has revolutionized E-healthcare technology up to the new heights of advancements [2] in order to fulfill the user’s expectations in the tele-operated mode of healthcare services. An E-healthcare CPS is generally a combination of a cyber-system of networks and physical system of sensors, medical equipment that provides the monitoring data to the practitioners /experts. This facility allows the patient to be observed from remote location by the medical practitioners where the data is observed either over a wireless network, wired network or mixed network. The diagram in Figure 1 shows an E-health CPS where practitioner monitors the health of their patient over the cyber space. Here, the health data from the equipment is transmitted through the cyber space to the practitioners at remote location. The cyber space consists of a network and an intelligent computational components where the whole working of CPS lies mostly on the intelligent component. However, the healthcare services are sometime critical and severely constrained with several parameters such as energy, risk, reliability, capacity and availability. Lots of researchers are seeking to strengthen the CPS. However, none of them have been concerned about their computational procedures. Recently, it has been found that the consumption of energy is a major issue for performing the computation and in CPS it is recommended to design an energy-efficient CPS. Furthermore, due to the environmental bar on energy resources, it is efficient to consume resources wisely. Therefore, research in CPS leads to green computations. Also, sometimes the working of CPS is found for critical services and when we are considering critical healthcare then it can be a matter of loss of life. Critical healthcare services are needed to be provided to the patient within requested service time (RST). The RST is the time for which a patient can survive, moreover, the RST of the patient also relies on the mean-time-to-failure (MTTF) of the services. The parameters discussed in the above paragraph need to be firmed up by providing agreements between service providers and users. These agreements are known as service-level-agreements (SLAs). Here, in the case of critical healthcare CPS, the SLAs are drawn in between practitioner and patient in terms of RST and MTTF of the service. These SLAs are the promise toward the satisfaction of services and provide the quality of service (QoS).

For the best explanation, there is a huge literature available to support the proposed system model and concepts. Although healthcare services are important requirements in society, sometimes risk of life loss is associated with the services. Therefore criticality constraint has to be applied. The continuity constraint to these applications is a very important parameter to fulfil the availability of energy requested to transmit the data between two specific ends. The healthcare data is sometime important and critical and must not be tempered; therefore, there is a need to propose some framework that provides a critical and energy-efficient CPS during the response of critical services. Furthermore, along with critical and energy-efficient CPS, security is considered to a great concern where patient health data is assumed to be confidential from ethical and legal perspectives. In the above paragraphs and literature, it has been analyzed that the discussed issues are highly sensitive toward the working of a CPS. Therefore, in this paper, we are considering the energy and SLA constraints collectively towards the proper functioning of CPS. Also, here we are assuming that our physical system is perfect and in functioning order. CPS has gradually becoming a widespread replacement technique with sensible cost-efficient emulation for connectivity to the family networking and community; it is officious to venture a proficient and safe communication mechanism. In CPS, security can be agreed simply because of its broadcasting, dynamic and distributed nature. Consequently, an ornate verification approach and a secure data transmission practice should be vital to promise that only legitimate devices have access to a variety of services with well-organized network recital. However, over these networks (wired/wireless), a patient’s data can be compromised due to the attacks. Therefore, special attention has been paid towards designing a secure architecture toward CPS. During the real-time transmission of messages, personal communication between patient and practitioner or storage of the patient’s report, there may be a possibility of an attack happening. A malicious node or a user may enter into an environment or hack one more legitimate node to behave maliciously where the attackers aim is to steal the communication between practitioner and patient, forge the patient’s reports, or perform some malicious activities in order to consume the network resources or slow down the communication process.

The proposal of the algorithm and mathematical formulation purely depends on the amount of healthcare data to be transmitted between two specific ends. The proposed mechanism follows end-to-end mechanism; therefore, the continuous maximum flow of healthcare data depends on the first and second equation. To deal with the workload, the maximum capacity has been considered at links. However, to deal with constant flow condition, the capacity of the path has been considered minimum with respect to the maximum healthcare data to be transmitted. Therefore, there is a need to propose some security mechanisms for an E-health CPS along with critical and energy efficient mechanisms. In order to ensure security during data transmission or personal communication between patient and practitioner or the storage of a patient’s report where malicious nodes or users are willing to disrupt the legitimate users or sensors, there is a need to deal with this issue.

The rest of the paper is structured into sections. Section 2 draws on the related work of the problem. Section 3 represents the proposed algorithms and preliminaries of the paper. Empirical analysis is carried out to highlight the theoretical results in Section 4. Section 5 is used for illustration of proposed algorithm and its time complexity. Experiment setup, results and discussion is given in Section 6. Finally, conclusion and future directions are given in the last section.

## 2. Related Work

With the passage of time, it has been seen that researchers from eminent research fields pioneered the powerful methods and tools to deal with the emerging CPS [3]. Development in physical systems improved CPS via advances in state space analysis, time and frequency domain analysis, tracking, optimization and filtering etc. Also, a number of scientists were concerned about the development of computational components technology with the design of new programming, body area sensors, biomedical sensors, computer system reliability, fault tolerance and cyber security. The below section discussed some of the energy SLA efficient, security frameworks needed to firm a strong basis for the development of proposed E-healthcare systems in a CPS.

The author in [4] discussed the importance of CPS in next-generation applications for the computing and integration of different applications such as transportation, health, manufacturing, energy and interdisciplinary applications. The functioning of CPS lies in three basic components sensing, computing/processing and networking [5]. The continued advances in wireless sensor networks (WSNs), medical sensors and reliable networks extended the involvement of CPS throughout in the field of E-healthcare and home-to-hospital (HTH) care or vice versa [6,7]. These applications became involved in body-area sensor networks and medical sensors, and therefore the research in this area became a hot topic [8,9]. Various researchers added their efforts to make these healthcare services easy and compatible using these sensors. However, it is difficult to manage wired sensor networks, and therefore the advancement of these sensors depends on the wireless sensor networks (WSNs) which gives the more comfort to the practitioner and patient. In addition to this, sometimes these healthcare services have been provided at remote locations via networking. This health data transmission has been requested as quickest with minimum delay. A number of authors [10,11,12,13,14,15] have been associated with the quickest path problem (QPP).

As this point, the health data of patient is helpful to provide necessary diagnostic /treatment/prescription to deal with the matter of patient life [16]. It is thanks to researchers that they have been provided with better solutions to deal with this compromising situation [17]. To add to this, while we deals with the wireless, wired or mixed sensor network then the health data over this are severely affected by the certain constraints [5] like energy, storage capacity, service level agreements, intelligent computing and processing etc. A number of authors have been associated with the different sections of the CPS [5,18]. However, lots of research has been underway in the field of networking to support the critical and continue health data transmission in the CPS [19].

In order to manage abnormal heart rate and cardiac diseases variability, authors in [20] have proposed a fractal technique for pacemaker design using a constrained horizon optimal controller issue. The proposed approach is achieved by moulding the dynamics of heart rate using fractional differential and calculus variations. Finally, along with practical implementation, researchers have discussed its hardware complexity. Also, authors in [21] have proposed an approach in order to facilitate the optimizing and designing of robust and efficient CPS for reducing diabetic costs in healthcare. The authors have designed a hardware model and proposed a mathematical model for amending the insulin injection problem for resolving the multi-fractal control issue. The accuracy of the proposed mathematical model is validated against existing non-fractal models. Later, in order to capture cross dependencies in spatial temporal fractal among united processes, authors have proposed a compact mathematical model. The proposed model is generalized and improved the accuracy for dynamic biological processes. Furthermore, the model is validated over certain medical observations [22]. In addition to this, authors in [23] have proposed a mathematical scheme for building accurate and compact complex system with the aim of scrutinizing influences and casual effects. In order to specify a single state at a time, the derived framework enables the incorporation of knowledge about inter-events and casual dynamics of magnitude increments. The presented framework permits us to examine the appropriateness of multi-fractional for various complex systems. The proposed approach validates the experimental results over various physiological processes against state of art techniques.

Networking abstractions to make the compatible CPS for healthcare are being developed [19] and lots of researchers are dealing with this [24,25,26]. Sometimes, healthcare services request reliable and promising health data transmission services [27,28,29]. Recently, it has been found that critical healthcare services relies more over the cyber component such as networking intelligent computing etc. Moreover, the health data is critical and there are requests for the reliable connection of networks [30,31,32]. A second delay in the services can lead to loss of life, and therefore for the need to design a health data transmission system without any violation in service level agreement [33,34]. For these types of services these SLAs plays a vital role in the support of CPS. The research in CPS shows the constraints of energy also; therefore, consideration of energy consumption can hold the computation of health data transmission [35]. Ignorance of energy constraints may interrupt services due to the lack of a sufficient amount of energy for health data transmission [36,37]. Also, due to deteriorating conditions of the environment and a bar on consumption of energy resources we are forced to consider these constraints on the data transmission [38]. In the networks, this confidential healthcare data of patient is requested to be made available to all concerned authorized medical personnel and, therefore, the chances of s security threat exist [39]. To tackle the crucial healthcare challenges, the authors have proposed a network on chip multi core platform for enabling the efficient molecular interaction among the entities. For analysing the interactions, communication and computation requirements, the authors have designed a high-performance network on chip (NoC) model that sustains a 1.36E5 events/ms throughput by consuming 15 mL energy per 1E5 stochastic events. The proposed approach offers 23% improvement with 20% less energy consumption against regular mesh NoC [40]. The authors in [41] have described two major fundamental challenges while designing a CPS framework for personalized healthcare systems. The need of a unifying mathematical description for designing CPS for providing dynamic interactions among cyber states and bio physiological events is considered as one major issue. Furthermore, the author has addressed secondary challenges for building a precise mathematical model for optimizing and designing wireless and wired NoCs.

Furthermore, a number of scientists and researchers have planned various safe routing approaches by defining several trusted and cryptographic based methods. For building the interaction from the outside world, a cyber-physical system must be reliable, efficient and secure. In order to optimize such systems, certain workload features such as non-stationary and self-similarity needed to be established. Authors have improved the CPS framework by enhancing the statistical approaches such as normalization group theory, master equations and fractional derivatives [42]. In [43], the authors proposed a feasible attack pattern mechanism against remote state estimation in CPS to analyze its corresponding effect on the performance metrics. To examine the optimal strategies for attacker and sensor, a game theoretic approach is built and the stability for mutually sides is deliberated. To identify the cyber-attack, the authors in [44] proposed a distributed multi-agent scheme over the protection systems of power grids. The malicious nodes on that protection system mimic legitimate faults and disable communication or cause component failure. The agents in the proposed mechanism employ both physical and cyber properties to strengthen the detection approach. The proposed approach is authorized through a benchmark power structure under several cyber-threat and fault scenarios. In order to explain and analyze the trustworthiness of cyber-physical measuring systems (CPMSs), generalized stochastic Petri nets are adopted by measuring against three metrics, i.e., availability, reliability and security in [45]. A malicious software spreading dynamics model is presented to learn about the trustworthiness evolution of CPMSs. The author in [46] proposed a service-oriented development approach for wide-area physical system such as vehicular networks and smart grid. Dissimilar to the traditional approach, the proposed methodology intrinsically permits disruption-free deployment. The proposed methodology broadens traditional service-oriented computing (SOC) concepts for managing real-time CPS features by pioneering QoS and resource-aware operation phases. The author in [47] presents a study of synthesis and analysis of the security and reliability of power CPS (PCPS). In this framework, the author considered the security management scenarios attained from the nature of each sort of cyber threat. The authors in [48] highlight industrial CPS security threats. The efficiency of the proposed scheme is verified by constructing an experimental fit. The simulated results reveal that the scheme deliberates a highly accurate solution that can effectively work in real-time scenarios. A number of secure approaches have been proposed for CPS against various applications such as industries, smart homes, and E-health. However, none of the proposed mechanism can provide the security in real-time scenarios with minimum delay, as time is also considered to be an important parameter while considering a E-health care CPS. A significant delay to ensure the security or legitimate the requested user allow number of intruders to analyse or consume the network resources. Therefore, along with a SLA critical and energy efficient mechanism, a secure E-healthcare CPS is needed to attract the users to rely on this application.

In literature, CPS and its applications have been discussed widely and rigorously. In this paper, the authors have tried to add the recent issues related to healthcare applications of CPS. The contribution can be seen as we have considered the energy constraint for the support of continuity of services for healthcare. This can be seen with the perspective of green computing which provides sustainable healthcare service of CPS. While we consider healthcare services, the assured services are the utmost requirement, and therefore another consideration can be seen by proposing SLAs for the healthcare data-transmission services. These SLAs are useful to support the critical healthcare application with the service assurance of CPS. In addition to this, sometimes healthcare data is confidential and, therefore, we have proposed a secure routing mechanism by doing some adjustment to the AODV protocol. The proposed security mechanism efficiently prevents the disruption of data packets during the transmission by addressing the security threat i.e., grey hole or black hole attack. The proposed method is confirmed against traditional routing mechanisms over several network metrics. The proposed approach is analyzed against the average number of s−t paths, mean hop counts for s−t path and mean energy efficiency. These results have been discussed for both cases without the involvement of a malicious device and with the involvement of a malicious device.

## 3. Proposed Framework

The proposed mechanism is discussed in number of steps along with their preliminaries:

- An energy-efficient E-healthcare CPS mechanism will be discussed for continuous healthcare data transmission;
- An SLA cooperative approach is defined to ensure a critical healthcare data transmission CPS mechanism;
- A secure E-health CPS will be defined as an extension for the discussed mechanism.

### 3.1. Preliminaries

In an E-healthcare CPS, the health data is transmitted from one end to other over a cyber-computer communication network (CCCN). A CCCN is modeled with the help of a graph such as G=(N,E), where N represents set of n nodes and E represents the set of m number of links. Every link of the network is assigned with specific link parameters such as capacity of link c(u,v) and delay of link d(u,v) [13,30]. A σ unit of data is transmitted between two consecutive nodes u and v by forming a link (u,v). The transmission time of a link is given by:

(1) $$T_{\sigma }\left(u,v\right)=d\left(u,v\right)+\lceil \frac{\sigma }{c\left(u,v\right)}\rceil$$

Data is transmitted along a path (P) between two specific ends source (s) to destination (t). For minimum transmission delay, the capacity of path is maximum, but for the flow network the capacity of the path is considered as minimum capacity of the path [30]. Therefore, the minimum transmission time is given by:

(2) $$T_{\sigma }\left(P\right)=\overset{i=k-1}{\underset{i=1}{\sum }}d\left(u_{i},u_{i+1}\right)+\lceil \frac{\sigma }{\min_{i=1}^{k-1}c\left(u_{i},u_{i+1}\right)}\rceil$$

Using Equation (2), the QPP model is given as:

(3) $$\begin{array}{c} \min T_{\sigma }\left(P\right)\\ s.t.P\;\mathrm{is}\;\mathrm{an}\;s-t\;\mathrm{path}\;\mathrm{in}\;\mathrm{network}\;G\left(N,E\right) \end{array}$$

The above model is useful to find the quickest path for data transmission. The problem has a great impact of data to be transmitted through a path (P). When the amount of data is small, then communication (transmission) time relies mainly on the delay factor of link and if the data is large then transmission time relies mainly on the path capacity (P). By using this model the proposed model is formulated in the next sub-sections.

### 3.2. Proposed Service-Level Agreement (SLA) System Model

In CCCN, for the continuity and criticality, all the link performance components are requested to be evaluated together in a single link parameter such as the service performance factor (SPF) [49] to find the qualifying service set (QSS). The following assumptions have been considered:

1. There are no parallel links or loop in the network to utilize the network resources efficiently.
2. Although nodes have been considered as perfect with respect to physical failure, these are subjected to the performance failure such as delay, traffic, requested SPF and bandwidth etc.

The SLA cooperation induced a great impact for completing the services in order to prevent network resources wastage [50]. During the problem formulation, these SLAs can be mapped in terms of RST $\left(t_{s}\right)$ and service MTTF_s_ in seconds, minutes, hours, weeks, months or years. In CCCN, the data unit’s communication services are occurred and completed in fraction of seconds, therefore, these are considered into seconds (s). As SPF is the part of link reliability, SLAs are requested to be modelled in such a way that it becomes comparable to the link reliability. Using the theory of reliability [51], the requested service performance factor (RSPF) at nodes denoted as $\left(r_{u}\right)$ is termed as the possibility of resilient service recital for a RST as defined in Equation (1) which is an important performance metric for getting the service.

(4) $$r_{u}=e^{-\frac{t_{s}}{MTTF_{s}}}$$

The completion of the data transmission process has been affected not only by the link reliability but also the delay, capacity and the amount of data transmitted through with it. For example, if any service experiences delay beyond the limit of obtaining the service. Then service MTTF_s_ can be considered as performance failure that further affects overall reliability of the link. Therefore, the integrity of all the link parameters is requested to make it comparable to link reliability. Its importance can be seen in a wide sense as it depends on the two essential factors (i) total transmission (communication) time and (ii) link of MTTF. As link delay, capacity, MTTF and data play a prominent role in the achievement of data transmission [49,52]; the mapping of SLAs has been incorporated in terms of service recital (performance) factor of link (SPF) denoted as $r_{s}\left(u,v\right)$ given by $r_{s}\left(u,v\right)=e^{-\left[\frac{d\left(u,v\right)+\frac{\sigma }{c\left(u,v\right)}}{MTTF\left(u,v\right)}\right]}$. Service performance factor of the path (*P*) is computed and expanded by putting Equation (2) and given as:

(5) $$R_{s}\left(P\right)=e^{-\left[\frac{\sum_{i=1}^{i=k-1}d\left(u_{i},u_{i+1}\right)+\lceil \frac{\sigma }{min_{i=1}^{k-1}\left[c\left(u_{i},u_{i+1}\right)\right]}\rceil }{\prod_{i=1}^{i=k-1}MTTF\left(u_{i},u_{i+1}\right)}\right]}\;⟹e^{-\left[\frac{\sum_{i=1}^{i=k-1}d\left(u_{i},u_{i+1}\right)}{\prod_{i=1}^{i=k-1}MTTF\left(u_{i},u_{i+1}\right)}\right]}\times e^{-\left[\frac{\left\lceil \frac{\sigma }{min_{i=1}^{k-1}\left[c\left(u_{i},u_{i+1}\right)\right]}\right\rceil }{\prod_{i=1}^{i=k-1}MTTF\left(u_{i},u_{i+1}\right)}\right]}\;⟹R_{s}\left(P\right)=\overset{k-1}{\underset{i=1}{\prod }}e^{-\left[\frac{d\left(u_{i},u_{i+1}\right)}{MTTF\left(u_{i},u_{i+1}\right)}\right]}\times e^{-\left[\frac{\left\lceil \frac{\sigma }{min_{i=1}^{k-1}\left[c\left(u_{i},u_{i+1}\right)\right]}\right\rceil }{\prod_{i=1}^{i=k-1}MTTF\left(u_{i},u_{i+1}\right)}\right]}\;⟹R_{s}\left(P\right)=e^{-\left[\frac{d\left(P\right)+\frac{\sigma }{c\left(u,v\right)}}{MTTF\left(P\right)}\right]}=e^{-\left[\frac{T_{\sigma }\left(P\right)}{MTTF\left(P\right)}\right]}\;∴\;R_{s}\left(P\right)=e^{-\left[\frac{T_{\sigma }\left(P\right)}{MTTF\left(P\right)}\right]}$$

In CCCN, there are number of nodes and links where each node can be a user, a service provider, a router or a computer. A path *P* is formed either combination of several links or a single link. Therefore, it is more realistic to satisfy the SLA piecewise or between two consecutive links other than satisfying the SLA after completion of the data transmission service among the path. The SLAs are considered for mission-critical applications; therefore, each node is endowed with the RSPF $\left(r_{u}\right)$ and relies as the possibility of resilient service for a particular service time $\left(t_{s}\right)$. Hence to satisfy criticality constraint across the link (u,v), SPF has to be more than that of RSPF given as:

(6) $$e^{\frac{-\left[d\left(u,v\right)+\frac{\sigma }{c\left(u,v\right)}\right]}{MTTF\left(u,v\right)}}\geq r_{u},\;\forall \left(u,v\right)\in E$$

The remaining SPF value is defined as residual requested service recital (performance) factor (RRSPF) along a path denoted as $r_{u}\left(\sigma ,P\right)$. The RRSPF is distinct as the residual endowed RSPF from the SPF at the nodes after entire message transmission beside a path. The role of the RRSPF has been used to locate the SLA supportive nodes which take part in the message transmission. In this paper, we use SLA cooperation and extensive reliability theory framework to improve the service recital. The RRSPF $r_{u}\left(\sigma ,P\right)$ along the path  P gives feasibility of path P i.e., $\;r_{u}\left(\sigma ,P\right)\geq 0,\;\forall \;u\in P$ as:

(7) $$r_{u}(\sigma ,P)=\{\begin{array}{cc} -\ln\left(r_{u}\right)-\left\{-\ln\left[e^{\frac{-\left[d\left(u_{i},u_{i+1}\;\right)+\frac{\sigma }{c\left(P\right)}\right]}{MTTF\left(u_{i},u_{i+1}\right)}}\right]\right\}, & \mathrm{if}\;u=u_{i},\;i=1,2,...,\;k-1\\ -\ln\left(r_{u}\right) & \mathrm{otherwise} \end{array}$$

The above equation is helpful for formulating the SLA cooperative quickest path problem for data transmission services:

(8) $$\begin{array}{c} \begin{array}{c} \underset{P}{\min}T_{\sigma }\left(P\right)\\ s.t.\;r_{u}\left(\sigma ,P\right)\geq 0,u\in P \end{array}\\ P\;\mathrm{is}\;\mathrm{an}\;s-t\;\mathrm{path}\;\mathrm{in}\;\mathrm{network}\;G \end{array}$$

In CCCN, plea energy at node u to transmit σ unit data over a link  (u,v) is called the energy rate  ω(u,v) and calculated as $\;\omega \left(u,v\right)\frac{\sigma }{c\left(u,v\right)}$. Since, a different number of nodes and links are present in the network, each node u∈N has been associated with the limited energy supply $\;\left(P_{u}\right)$ provided with batteries [37]. To satisfy the continuity, σ data transmission through link (u,v) has to be more than associated energy supply with requested energy (RE) given as:

(9) $$P_{u}\geq \omega \left(u,v\right)\frac{\sigma }{c\left(u,v\right)},\;\forall \left(u,v\right)\in E$$

The rest of the leftover value is defined as residual energy supply (RES) along a path $P_{u}\left(\sigma ,P\right)$. RES is termed as the rest artistic energy supply from the plead energy at the nodes after complete data transfer along a path. RES used to locate the energy supportive nodes which take part in the data transfer [36]. The RES $P_{u}\left(\sigma ,P\right)$ along the path  P gives the feasibility of path  P i.e., $\;P_{u}\left(\sigma ,P\right)\geq 0,\;\forall \;u\in P$ as:

(10) $$P_{u}(\sigma ,P)=\{\begin{array}{ll} P_{u}-\omega \left(u_{i},u_{i+1}\;\right)\frac{\sigma }{c\left(P\right)}, & if\;u=u_{i},\;i=1,...,\;k-1\\ P_{u} & \mathrm{otherwise} \end{array}$$

Using the above equation, the energy cooperative quickest path dilemma is formulated as:

(11) $$\begin{array}{c} \begin{array}{c} \underset{P}{\min}T_{\sigma }\left(P\right)\\ s.t.\;P_{u}\left(\sigma ,P\right)\geq 0,u\in P \end{array}\\ P\;\mathrm{is}\;\mathrm{an}\;s-t\;\mathrm{path}\;\mathrm{in}\;\mathrm{network}\;G \end{array}$$

Using Equations (11) and (12), the quickest path problem model is capable for SLA and energy satisfied QPP for data transmission given as:

(12) $$\begin{array}{c} \begin{array}{c} \underset{P}{\min\;}T_{\sigma }\left(P\right)\\ \begin{array}{c} s.t.\;\;\;r_{u}\left(\sigma ,P\right)\geq 0,\;\;u\in P\\ P_{u}\left(\sigma ,P\right)\geq 0,\;\;u\in P \end{array} \end{array}\\ \;\;\;\;\;\;\;\;\;\;\;\;\;\;\;P\;\mathrm{is}\;\mathrm{an}\;s-t\;\mathrm{path}\;\mathrm{in}\;\mathrm{network}\;G \end{array}$$

### 3.3. Secure Cyber-Physical System (CPS) Framework

**Figure 2:**
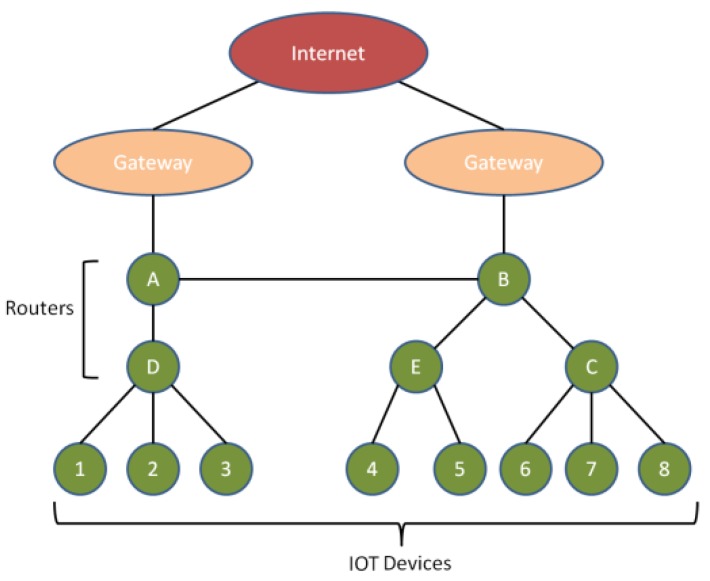
Data flow in cyber-physical system (CPS) network.

Figure 2 presents a CPS network having a number of IoT devices, sensor nodes, routers, gateways and internet.

**Figure 3:**
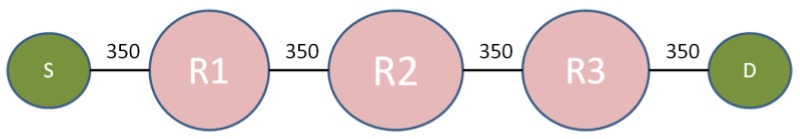
Data transmission in CPS.

The routers and gateways are generally are static and provide communication among the devices. The architecture of CPS is hierarchical in nature; the top layer comprises the internet to provide the services to users. Routers are the intermediate level through which services are provided. IoT devices constitute the lowest layer that utilize the real-time network services. In order to understand the entire working of proposed framework, let us believe a situation where foundation (source) node ‘S’ needs to communicate with destination node ‘D’ as depicted in Figure 3.

Let ‘S’ propels the data to ‘D’ through R1-R2-R3 path, in order to ensure the legitimacy of intermediate nodes every node will compute the legality of its previous node via previous node validity (PNV).

$$PNV=\frac{Message\;received\;by\;current\;node}{Message\;recived\;by\;previous\;node}$$

If PNV satisfies threshold ratio, previous node is legitimate else the present node will propel as alarm memo to its 2-hop previous node to alert the node to reroute the data packet. Let ‘S’ conveys 350 packets to ‘D’, each node forwards all packet if they are legitimate. If a node accepts less packets then there may be possibility of grey hole or black hole attacks. If the number of packets inward by the present node is less than 75%, then there may be a chance of grey hole or black hole attacks. A black hole attack drops all the services transmitted between source and destination while a grey hole attack selectively drops the services making it crucial to identify it in initial stages. The network metrics severely affect the proposed framework that is why we have taken these two attacks only. The current node will instantly send alerts to its 2-hop previous node to stop further transmission of messages to that path.

#### 3.3.1. Case 1: Without Contribution of Malicious Device

If ‘S’ sends 350 packets to R1 and R1 received all 350 packets and forwards to R2, the R1 and R2 will compute the PNV as given in the following equation:

$$Node\;S=\frac{350\;\left(Node\;1\right)}{350\;\left(Node\;S\right)}$$

Similarly, $Node\;1=\frac{350\;\left(Node\;2\right)}{350\;\left(Node\;1\right)}$.

The PNV of ‘S’ is 1 means ‘S’ and R1 both are legitimate. Similarly, all the nodes will check the legitimacy of their preceding nodes by computing PNV.

#### 3.3.2. Case 2: With Participation of Malevolent Device

**Figure 4:**
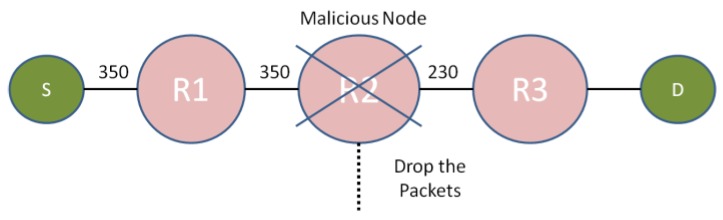
Data transmission in CPS in the existence of a malicious node.

Let node R2 is malevolent. ‘S’ sent 350 packets to R1 as depicted in Figure 4. As R1 is legitimate, it will send all 350 packets to R2. Now, R2 is malicious nodes, therefore, R2 intentionally drop some packets and forward intentionally drop some packets to its succeeding node i.e., R3. Now, node R3 will compute the PNV value of R2 as:

$$R2_{PNV}=\frac{230\;\left(Node\;3\right)}{350\;\left(Node\;2\right)}$$

As the PNV of R2 is less than the threshold, this means R2 is malicious. In order to further confirm whether the dropping of packets are due to congestion or malicious node, R3 overhears its 2-hop preceding node i.e., R1 and check its PNV value. If the PNV value of R1 is more than R2 then it will immediately alert the R1 is more than R2 then it will immediate alert the R1 to reroute its data packets to any other nodes using AODV algorithm and declare the R2 as malicious nodes.

## 4. Empirical Analysis

Generally, there are r different capacities $c_{1}<c_{2}<\ldots <c_{r}$ present in any CCCN. The minimum energy and SLA cooperative link capacity is given below in Equations (13) and (14), and corresponding to this the minimum SLA and energy supportive link capacity is also revealed as in Equation (15):

(13) $$c_{minSLA}\left(u,v\right)=\underset{i=1,...,\;r}{\min}\left\{c_{i}\;\;:\;\;e^{\frac{-\left[d\left(u,v\right)+\frac{\sigma }{c_{i}}\right]}{MTTF\left(u,v\right)}}-r_{u}\;\geq 0\right\}$$

(14) $$c_{minE}\left(u,v\right)=\underset{i=1,...,\;r}{\min}\left\{c_{i}\;\;:\;\;\;P_{u}-\omega \left(u,v\right)\frac{\sigma }{c_{i}}\geq 0\right\}$$

The Equation (14), helps to corporate the efficient use of energy for continues data transmission. Therefore, the procedure has been given as follows for the support of continuity.

(15) $$c_{\min}\left(u,v\right)=\min\left\{\left[c_{minE}\left(u,v\right)\geq c_{a}\geq c\left(u,v\right)\right]\cap \left[c_{minSLA}\left(u,v\right)\geq c_{b}\geq c\left(u,v\right)\right]\right\}$$

where $c_{a}$ and $c_{b}$ are the competence lies in the minimum supportive energy and SLA ability, respectively and link capacity Equation (15) provides the label of least link capacity to hold the criticality and continuity in message transmission if $\;c_{minSLA}\left(u,v\right)$ and $c_{minE}\left(u,v\right)>0$. A  s−t path P is feasible if $c\left(P\right)\geq c_{min}\left(u,v\right)$. The above equations kind the least capacity which integrates both continues and critical data transmission allowing for the AND rule. The AND rule is mentioned here as for a precise link together parameters Energy and SLA needs to be satisfied. Let us assume that when a link chains several parameters then the logic has been given it as “1” otherwise “0”. Now using possessions of AND gate, the link will support the least capacity $c_{min}\left(u,v\right)$ only when both parameter gives logic “1”. Therefore, $c_{min}\left(u,v\right)$ has to trail the AND rule for secure, energy and SLA-efficient healthcare message transmission for task-critical relevance. From Equation (15), r number of sub-networks has been sort and every link has to pursue the given variation for the path capacity  c(P).

(16) $$c\left(u,v\right)\geq c_{j}\geq c_{min}\left(u,v\right)\;;where\;j=1,2,\;\ldots ,r$$

**Lemma** **1.** *Suppose a path* $P=u_{1},u_{2}\;\ldots ,\;u_{k-1},\;u_{k}$ *has been recognized as the* s−t *path in a sub-network then that path has been identified as SLA- and energy-cooperative (SESE).*

**Proof.**  Path P is s−t path in the sub-network, the path capacity c(P) has to follow $\;c\left(P\right)\geq c_{j}\geq c_{min}\left(u_{i},u_{i+1}\right)$, where  i=1,…,k−1. Hence:

$$P_{u}\left(\sigma ,P\right)=P_{u}-\omega \left(u_{i},u_{i+1}\right)\frac{\sigma }{c\left(P\right)}\geq P_{u}-\omega \left(u_{i},u_{i+1}\right)\frac{\sigma }{c_{min}\left(u_{i},u_{i+1}\right)}\geq 0$$

$$r_{u}\left(\sigma ,P\right)=e^{\frac{-\left[d\left(u_{i},u_{i+1}\right)+\frac{\sigma }{c\left(P\right)}\right]}{MTTF\left(u_{i},u_{i+1}\right)}}-r_{u}\geq e^{\frac{-\left[d\left(u_{i},u_{i+1}\right)+\frac{\sigma }{c_{min}\left(u_{i},u_{i+1}\right)}\right]}{MTTF\left(u_{i},u_{i+1}\right)}}-r_{u}\geq 0$$

□

**Lemma** **2.** *Let a*s−t *path*P *is thought to be a possible path having capacity of path*$c\;\left(P\right)=c_{j}$*, then*P*is a path in*$G_{j}$.

**Proof.**  From above Lemma 1, let P is possible.

$$P_{u}\left(\sigma ,P\right)=P_{u}-\omega \left(u_{i},u_{i+1}\right)\frac{\sigma }{c\left(P\right)}\geq 0,i=1,\;2,\;\ldots ,\;k-1.\;$$

$$r_{u}\left(\sigma ,P\right)=e^{\frac{-\left[d\left(u_{i},u_{i+1}\right)+\frac{\sigma }{c\left(P\right)}\right]}{MTTF\left(u_{i},u_{i+1}\right)}}-r_{u}\geq 0,\;i=1,\;2,\;\ldots ,\;k-1$$

□

By satisfying Equation (16), the path (P) is s−t path in network $G_{j}$. The computation of path (P) depends on the shortest path problem (SPP) which follows Dijkastra’s algorithm. The computation of the path depends on the cost function which is taken as link delays i.e., d(u,v).

(17) $$\begin{array}{c} {\mathrm{SPP}}_{j}\;\;\;:\;\;\;\;\underset{P}{\min}d\left(P\right)\\ s.t.\;\;P\;\mathrm{is}\;a\;s-t\;\mathrm{path}\;\mathrm{in}\;\mathrm{the}\;\mathrm{network}\;G_{j} \end{array}$$

After Equation (17), Lemma 3 needs to be explained as below:

**Lemma** **3.** *Given,* P *is a most favorable path computed by* $SPP_{j}$ *given that* $c\left(P\right)=c_{h}>c_{j}$ *. In that case, no other most favorable path is there for algorithm SESE having capacity* $c_{j}$ *.*

**Proof.**  Take; Q as a s−t possible path for the algorithm SESE having capacity $\;c_{j}$, then Q is a path in $\;G_{j}$.

$$T_{\sigma }\left(P\right)=d\left(P\right)+\frac{\sigma }{c_{h}}<d\left(Q\right)+\frac{\sigma }{c_{j}}=T_{\sigma }\left(Q\right)$$

Hence, Q cannot be a most favorable path for the algorithm SESE. □

**Theorem** **1.** *Consider*$\overset{ˇ}{P}$*be a most favorable path for SESE and*$\left(\overset{ˇ}{P}\right)=c_{h}$*. Then,*$\overset{ˇ}{P}$*is a most favorable path of*$SPP_{h}$*and any most favorable path of*$SPP_{h}$*is a most favorable path*.

**Proof.**  Given that $\overset{ˇ}{P}$ is an s−t possible path for SESE having capacity $\;c_{h}$, then $\overset{ˇ}{P}$ is an s−t path in $G_{h}$. Consider Q is a s−t possible path in network $\;G_{h}$, then $\;c\left(Q\right)\geq c_{h}$. Also, if $\;d\left(Q\right)<d\left(\overset{ˇ}{P}\right)$, then

$$T_{\sigma }\left(Q\right)=d\left(Q\right)+\frac{\sigma }{c\left(Q\right)}<d\left(\overset{ˇ}{P}\right)+\frac{\sigma }{c_{h}}=T_{\sigma }\left(\overset{ˇ}{P}\right)$$

which disagree with the condition of most favorable path $\overset{ˇ}{P}$. In addition to this, using Lemma 3, capacity of s−t shortest path $\tilde{P}$ in $G_{h}$ is $c\left(\tilde{P}\right)=c_{h}$. Hence, $\tilde{P}$ is a s−t possible path for SESE such that $T_{\sigma }\left(\tilde{P}\right)=T_{\sigma }\left(\overset{ˇ}{P}\right)$ is the most favorable path. □

## 5. Proposed Algorithms and Algorithmic Time Complexity

### 5.1. Algorithm 1


**Algorithm 1: Secure, Energy- and SLA-Efficient (SESE) Algorithm without involvement of malicious device**
Input: $\;G\left(N,E\right),\sigma ,\;d\left(u,v\right),\;\omega \left(u,v\right),c\left(u,v\right)\;,\;MTTF\left(u,v\right),\;r_{u},P_{u}t_{s}\;\mathrm{and}\;MTTF_{s}$
Output: SLA-energy Cooperative Quickest Path (SESE)BEGIN{Initialization:
j←1, 
Procedure:STEP 0: Variable Declaration
G← Network

N←Set of nodes

E←Set of links

c(u,v)←Capacity of the link (u,v)

d(u,v)←Delay of link the (u,v)

ω(u,v)←Energy rate of link the (u,v)

$P_{u}\leftarrow \mathrm{Endowed}\;\mathrm{energy}\;\mathrm{at}\;\mathrm{node}\;u$

$r_{u}\leftarrow \mathrm{Requested}\;\mathrm{service}\;\mathrm{performance}\;\mathrm{factor}\;\mathrm{at}\;\mathrm{node}\;u$

σ←Data

$t_{s}\leftarrow \mathrm{Requested}\;\mathrm{service}\;\mathrm{time}$

$MTTF_{s}\leftarrow \mathrm{Mean}\;\mathrm{Time}\;\mathrm{to}\;\mathrm{Failure}\;\mathrm{of}\;\mathrm{service}$

MTTF(u,v)←Mean Time to Failure of a link
STEP 1: Find r capacities corresponds to critical-continuous service and label of minimum capacity:(i)    $c_{1}<c_{2}<c_{3}\cdots <c_{r}$(ii)   $c_{min}\left(u,v\right)$ with AND ruleSTEP 2: Solve $SPP_{j}$ w.r.t. delay time d(u,v) in $G_{j}$ with capacities $c_{j}$
For  j←1:r
   Set j←1
   Solve $SPP_{j}$.     If No s−t path in $G_{j}$ with capacity with $c_{j}$
       go to STEP3     else       Let $P_{j}$ is an optimal solution for ${\mathrm{SPP}}_{j}$ with capacity $c\left(P_{j}\right)=c_{j}$
     end  endSTEP 3:If j= r
     go to STEP4else     set j=j+1 and go to STEP2endSTEP 4: Find the solutionCompute the index h∈(1, 2,…r) of path array $P_{j}$

$T_{\sigma }\left(P_{h}\right)=\underset{j=1,\ldots ,\;\;r}{\min}T_{\sigma }\left(P_{j}\right)$
$P_{h}$ is an optimal solution of the SESE} END

### 5.2. Algorithm 2


**Algorithm 2: Secure, Energy- and SLA-Efficient (SESE) Algorithm with involvement of malicious device**
Input: $\;G\left(N,E\right),\sigma ,\;d\left(u,v\right),\;\omega \left(u,v\right),c\left(u,v\right),\;P_{u},MTTF\left(u,v\right),\;r_{u},t_{s}\;\mathrm{and}\;MTTF_{s}$
Output: Secured SLA-energy Cooperative Quickest Path (SESE)BEGIN{Initialization:
j←1, 
Procedure:STEP 0: Variable Declaration
G←Network

N←Set of nodes

E←Set of links

c(u,v)←Capacity of the link (u,v)

d(u,v)←Delay of link the (u,v)

ω(u,v)←Energy rate of link the (u,v)

$P_{u}\leftarrow \mathrm{Endowed}\;\mathrm{energy}\;\mathrm{at}\;\mathrm{node}\;u$

$r_{u}\leftarrow \mathrm{Requested}\;\mathrm{service}\;\mathrm{performance}\;\mathrm{factor}\;\mathrm{at}\;\mathrm{node}\;u$

σ←Data

$t_{s}\leftarrow \mathrm{Requested}\;\mathrm{service}\;\mathrm{time}$

$MTTF_{s}\leftarrow \mathrm{Mean}\;\mathrm{Time}\;\mathrm{to}\;\mathrm{Failure}\;\mathrm{of}\;\mathrm{service}$

MTTF(u,v)←Mean Time to Failure of a link
STEP 1: Find r capacities corresponds to critical-continuous service and label of minimum capacity:(iii)    $c_{1}<c_{2}<c_{3}\cdots <c_{r}$(iv)   $c_{min}\left(u,v\right)$ with AND ruleSTEP 2: Solve $SPP_{j}$ w.r.t. delay time d(u,v) in $G_{j}$ with capacities $c_{j}$
For  j←1:r
   Set j←1
   Solve $SPP_{j}$
     If No s−t path in $G_{j}$ with capacity with $c_{j}$
       go to STEP3     else       Let $P_{j}$ is an optimal solution for ${\mathrm{SPP}}_{j}$ with capacity $c\left(P_{j}\right)=c_{j}$
     end  endSTEP 3:If j= r
     go to STEP4else     set j=j+1 and go to STEP2endSTEP 4: Find the legitimate nodeIf PNV<75%
  Remove node from the networkelsego to STEP 5STEP 5: Find the solutionCompute the index h∈(1, 2,…r) of path array $P_{j}$

$T_{\sigma }\left(P_{h}\right)=\underset{j=1,\ldots ,\;\;r}{\min}T_{\sigma }\left(P_{j}\right)$
$P_{h}$ is an optimal solution of the SESE} END

### 5.3. Algorithm Time Complexity

**Theorem** **2.** *The proposed SESE algorithm has time complexity of*  O(r(m+n(log(n))) *and space complexity.*

**Proof.**  The complexity of the proposed algorithm heavily relies on Dijkstra’s algorithm [53] which has the time complexity of O(m+n log(n)) where (m) is the set of number of links and (n) is the set of nodes with O(m+n) space complexity. Here, the proposed algorithm has been run for (r) number of distinct capacities and gives a shortest path using Dijkstra’s algorithm. Therefore, the time and space complexity of the proposed algorithm is given by O(r(m+n log(n))) and O(n+m), respectively. □

## 6. Simulation Results and Discussion

### 6.1. Experiment Setup

The experiment was conducted on a personal computer with Intel(R) Core^TM^i5–7400, CPU@ 3.00 GHz, 8-GB RAM manufactured by Dell, and Windows 10 operating system in MATLAB 2010a. The SESE algorithm included calculation of the path utilizing the AODV calculation. The extent of the projected model was reenacted utilizing hop count, qualifying administration set of paths and vitality productivity. For the understanding the pertinence and convenience of the proposed algorithm, the outcomes have been introduced for the arbitrary systems produced by a Waxman irregular topology generator.

**Waxman Random Topology Generator:**

To examine the execution of the projected SESE algorithm on the extensive random network systems, a Waxman arbitrary topology producer was utilized [54,55]. The generation of Waxman topology are finished by setting the nodes in a one-by-one square, and the connections are made among two nodes (u) and (v) by thinking about the likelihood possibility.

(18) $$P\left(u,v\right)=\alpha e^{-\left(\frac{d\left(u,v\right)}{\beta L}\right)}$$

where: d(u,v) is the Euclidean distances between (u) and (v), α is the greatest link probability such that α>0, β is the attribute to control length links, L is the greatest separation among any two connections. The distinctive estimations of α and β were measured as 0.4 and 0.1, separately. To upgrade the lucidity of results, distinctive estimations of information traffic, vitality and SLAs have been considered. The projected algorithm was confirmed for various arrangements of network measurements, as depicted in Table 1, for example, number of links, nodes energy, information SLAs and traffic. These qualities are appeared beneath:

The distinct network recital attributes are worn to evaluate the performance for the algorithm. The network attributes are used as average contender s−t QSS paths, mean hop-count and mean energy efficiency. The mean applicant s−t QSS paths are the parameter for getting the average number of candidate ideal s−t consistent and speediest paths for the information system. Mean hop count is the execution assess for ascertaining the energy effectiveness. On the off chance that the quantities of average hop count is decremented, then mean energy effectiveness is incremented. The energy effectiveness is the execution measure for proficient utilization of energy for information transmission administrations or services and it is measured as the measure of information traffic exchanged to the absolute energy devoured for the information transmission over the s−t paths. The units for the energy efficiency are measured in terms of bits/secs/joule. Here, the amount of data has been considered in Mb, therefore here the units for energy efficiency are considered as Mb/secs/joule.

### 6.2. Results and Discussion

#### 6.2.1. Without Attack

The first, second and third segments of Table 2, Table 3 and Table 4 demonstrate the variety of various number of capacities, nodes and connections related with the network systems, separately. The variety in the estimations of the mean number of applicable s−t QSS paths, hop count and mean vitality effectiveness are shown in the following successive segments of Table 2, Table 3 and Table 4 for the information esteems 1 Mb, individually. These results show here are with the 95% confidence region or with 5% error.

The above observation gives a brief idea of the importance of the algorithm with reference to energy and SLAs to support significant healthcare applications. The dissimilarity of SLAs and energy is also a vital factor to support all these services. The performance of the performance attributes decreases if there is in increase in the data payload. One can increment the SLAs and energy attribute values to achieve high data transfer value, which ultimately is more favorable for the continuity of service. Also, for greater understanding, the experiment has been conducted for 10 Mb data transmission also as depicted in Table 5, Table 6 and Table 7.

For the critical service, whose principal constraints cannot be compromised over other conditions, other media have to be used for the completion of the service, such as green corridors, dedicated networks etc. In the next section we are showing our results with occurrence of malicious nodes in the network where healthcare data can be compromised.

#### 6.2.2. With Attack

In this section, results have been illustrated with the help of a random network generator, where nodes are attacked and become malicious to the network. These malicious nodes compromised the data of a patient. Therefore, by proposing a security enabled framework for the healthcare data, the patient’s data cannot be compromised. The results during attack are analyzed in Table 8, Table 9, Table 10, Table 11, Table 12 and Table 13.

While comparing the simulated results in both the scenarios such as with attack and without attack, it has been seen that the trend of network resources experiences some degradation. This scenario is tolerable at the cost of security of healthcare data of a patient which is more important.

Let us analyze the results quantitatively with the Table 2 column 4 and Table 8 column 4 values, whereby the trends show that for 10 distinct capacities and 100 nodes the average QSS paths decrease when attacks occurred in the network. This trend has been occurred because of the removal of malicious network resources at the cost of security. Furthermore, from Table 3 and Table 9 with column 4, the results for the hop counts for the 10 distinct capacities having 100 nodes shows that number of hopes increased. However, the hope count has not had such a big effect because network nodes have a sufficient degree to become connected after being generated via a Waxman network generator. The attacks in the network made a change in its energy efficiency too and, therefore, from Table 4 and Table 10 with column 4 this can be easily analyzed qualitatively as well as quantitatively. Same variation pattern has been analyzed for the 10 *Mb* data transmission in Table 5, Table 6 and Table 7 without attack and Table 11, Table 12 and Table 13 with attack.

## 7. Conclusions and Future Scope

In this paper, a secure and SLA- and energy-efficient healthcare CPS is proposed that can professionally secure the communication procedure, reports between practitioner and patient, and respond to the user’s request with minimum delay. By identifying the residual information of energy at the node before the transmission process, prior knowledge of requested service time and modification in AODV process mechanisms efficiently provide a secure and efficient communication mechanism. With and without involvement of malicious nodes, the simulated analysis of the proposed framework against average hop count, average energy efficient and mean quickest paths parameters outperforms conventional approaches. Furthermore, the results of SLA and energy vary over these parameters in order to analyze the pattern that governs an important aspect for considering an efficient path selection in the communication process. Moreover, the measured quantitative and qualitative results perform efficiently without the contribution of malicious devices, while during the contribution of malicious nodes the performance degrades at the cost of security. In our future communications, we deliberate to analyze the proposed mechanism over further security attacks i.e., byzantine, jelly fish and worm hole attacks and try to reduce the data size for efficiently utilizing the network resources.

## Figures and Tables

**Table 1:** Estimations of several network metrics for arbitrary experiment.

Sr. No.	List of Network Attributes	Attribute Values
1	Total nodes	100, 200 and 300
2	Total links	4600, 18,500 and 41,500
3	Total number of different capacities	10,100 and 1000
4	Information traffic	1, 10 *Mb*
5	Distinct energy allied with nodes	10, 100 and 100 *joule*
6	Distinct SLAs assigned	100, 110 and 120 *secs*

**Table 2:** Average candidate *s* − *t* qualifying service set (QSS) paths for SESE algorithm for 1 *Mb*.

*r*	*n*	*m*	Average Candidate s − t QSS Paths for 10 Runs								
			*P_u_* = 10			*P_u_* = 100			*P_u_* = 1000		
			*t_s_* = 100	*t_s_* = 105	*t_s_* = 110	*t_s_* = 100	*t_s_* = 105	*t_s_* = 110	*t_s_* = 100	*t_s_* = 105	*t_s_* = 110
10	100	4600	6.4	8.5	8.7	8.9	9.4	9.5	9.1	9.5	9.8
	200	18,500	7.8	9.2	9.2	9.3	9.6	9.4	9.3	9.4	9.4
	400	74,000	8.4	8.8	9.2	9	9.4	9.8	9.2	9.4	9.6
100	100	4600	38	52.7	54	56.6	61	62.6	58.5	59.9	60.7
	200	18,500	49.8	56.6	56.7	58.5	60.8	61.6	59.1	61.8	62
	400	74,000	60	60.5	62	64.5	62.5	62.5	88	92.4	94.2
1000	100	4600	63.9	80.6	82.6	89.6	95.7	95.3	90.5	95.6	96.2
	200	18,500	72.6	89.6	90	92.8	96.4	97.6	94.2	97.4	98
	400	74,000	88.5	90.6	91	92.4	94	94.3	95.1	96.8	98

**Table 3:** Mean hop counts for the effective path for the SESE algorithm for 1 *Mb*.

*r*	*n*	*m*	Mean Hop Counts for the Effective Path for 10 Runs								
			*P_u_* = 10			*P_u_* = 100			*P_u_* = 1000		
			*t_s_* = 100	*t_s_* = 105	*t_s_* = 110	*t_s_* = 100	*t_s_* = 105	*t_s_* = 110	*t_s_* = 100	*t_s_* = 105	*t_s_* = 110
10	100	4600	2.8	2.7	2.8	2.5	2.7	3.6	2.9	2.3	2.9
	200	18,500	3	2.8	2.5	2.6	2.5	2.4	2.7	2.5	2.3
	400	74,000	3.6	3.4	3.2	3.2	3.4	3.4	3	3	2
100	100	4600	4.1	2.7	2.6	2.9	2.6	2.6	2.9	2.6	2.5
	200	18,500	3.2	3.1	2.9	3.6	3	2.8	3.1	3	3
	400	74,000	4.5	4	3.5	3	2.5	2	4.5	3	2.8
1000	100	4600	2.5	2.4	2.3	3.2	2.7	2.5	3.2	2.3	2.2
	200	18,500	2.8	2.4	2.6	3.6	2.8	2.6	3.6	2.8	2.6
	400	74,000	3.9	3.8	2.5	3.3	3	2.8	3.6	3.2	3.2

**Table 4:** Mean energy efficiency for the effective path for the SESE algorithm for 1 *Mb*.

*r*	*n*	*m*	Mean Energy Efficiency for the Effective Path for 10 Runs								
			*P_u_* = 10			*P_u_* = 100			*P_u_* = 1000		
			*t_s_* = 100	*t_s_* = 105	*t_s_* = 110	*t_s_* = 100	*t_s_* = 105	*t_s_* = 110	*t_s_* = 100	*t_s_* = 105	*t_s_* = 110
10	100	4600	0.36333	0.90749	1.22174	0.39798	1.0771	1.19586	0.3498	1.9055	1.9706
	200	18,500	1.05942	2.01246	4.28012	1.75267	1.82279	7.35506	0.4624	1.69919	3.94239
	400	74,000	1.00162	3.3933	4.60966	3.82324	4.5407	6.28248	2.5712	5.707	5.744
100	100	4600	0.44407	0.51382	1.04791	0.55043	0.58404	1.53078	0.6695	0.84102	1.20749
	200	18,500	0.90359	1.3701	1.68088	1.34248	2.03887	2.49683	2.2992	2.12448	2.6691
	400	74,000	0.40265	1.3386	5.5728	2.99975	2.36895	3.74615	1.5138	3.5489	4.3569
1000	100	4600	2.66702	1.27768	1.51618	0.54678	1.13749	1.26496	0.4201	1.83122	2.67396
	200	18,500	0.4552	2.03994	2.6679	0.6947	1.6202	2.7475	0.8940	0.92748	1.39722
	400	74,000	1.5689	2.0695	4.3663	0.6875	1.5423	3.2595	0.3624	0.5983	1.7999

**Table 5:** The average candidate s − t QSS paths for the SESE algorithm for 10 Mb.

*r*	*n*	*m*	Average Candidate s − t QSS Paths for 10 Runs								
			*P_u_* = 10			*P_u_* = 100			*P_u_* = 1000		
			*t_s_* = 100	*t_s_* = 105	*t_s_* = 110	*t_s_* = 100	*t_s_* = 105	*t_s_* = 110	*t_s_* = 100	*t_s_* = 105	*t_s_* = 110
10	100	4600	4.5	6.6	6.8	7.1	7.5	7.6	7.2	7.6	7.9
	200	18,500	5.9	7.3	7.3	7.4	7.7	7.6	7.4	7.5	7.5
	400	74,000	6.5	6.9	7.3	7	7.5	7.9	7.3	7.5	7.7
100	100	4600	36	50.8	52	54.5	59	60.7	56.6	58.1	58.8
	200	18,500	47.9	54.6	54.8	56.6	58.9	61.6	57.2	59.9	60
	400	74,000	58	58.6	60	62.5	60.6	60.6	86	90.6	92.3
1000	100	4600	62.1	78.7	80.7	87.7	93.8	93.4	88.6	93.7	94.3
	200	18,500	70.7	87.7	88	90.9	94.6	95.7	92.4	95.6	96
	400	74,000	86.6	88.7	89	90.5	92	92.4	93.2	94.9	96

**Table 6:** Mean hop counts for the effective path for the SESE algorithm for 10 *Mb*.

*r*	*n*	*m*	Mean Hop Counts for the Optimal Path for 10 Runs								
			*P_u_* = 10			*P_u_* = 100			*P_u_* = 1000		
			*t_s_* = 100	*t_s_* = 105	*t_s_* = 110	*t_s_* = 100	*t_s_* = 105	*t_s_* = 110	*t_s_* = 100	*t_s_* = 105	*t_s_* = 110
10	100	4600	2.4	2.3	2.4	2.1	2.3	3.2	2.5	2	2.5
	200	18,500	2.6	2.4	2.1	2.2	2.1	2	2.3	2.1	2
	400	74,000	3.2	3	2.8	2.8	3	3	2.6	2.6	1.6
100	100	4600	3.7	2.3	2.2	2.5	2.2	2.2	2.5	2.2	2.1
	200	18,500	2.8	2.7	2.5	3.1	2.6	2.4	2.7	2.6	2.6
	400	74,000	4.1	3.6	3.1	2.6	2.1	1.6	4.1	2.6	2.4
1000	100	4600	2.1	2	1.9	2.8	2.3	2.1	2.8	1.9	1.8
	200	18,500	2.4	2	2.2	3.2	2.4	2.2	3.2	2.4	2.2
	400	74,000	3.5	3.4	2.1	2.9	2.6	2.4	3.2	2.8	2.8

**Table 7:** Mean energy efficiency for the effective path for the SESE algorithm for 10 *Mb*.

*r*	*n*	*m*	Mean Energy Efficiency for the Optimal Path for 10 Runs								
			*P_u_* = 10			*P_u_* = 100			*P_u_* = 1000		
			*t_s_* = 100	*t_s_* = 105	*t_s_* = 110	*t_s_* = 100	*t_s_* = 105	*t_s_* = 110	*t_s_* = 100	*t_s_* = 105	*t_s_* = 110
10	100	4600	0.33666	0.87759	1.19175	0.36799	1.0477	1.15666	0.3187	1.8745	1.9486
	200	18,500	1.01943	2.04246	4.24013	1.71254	1.788	7.3166	0.4227	1.65987	3.9046
	400	74,000	1.04162	3.3456	4.54045	3.78564	4.50989	5.24245	1.9712	4.7087	4.7542
100	100	4600	0.5942	0.61892	1.2791	0.89049	0.90404	1.95088	0.7595	0.98155	1.8974
	200	18,500	0.9535	1.3807	1.72056	1.38265	2.07845	2.54456	2.3598	2.1648	2.781
	400	74,000	0.5425	1.5486	5.7829	3.09977	2.56845	3.95617	1.7537	3.7588	4.5599
1000	100	4600	2.86707	1.45767	1.7265	0.74677	1.34748	1.49497	0.6468	1.98127	2.8442
	200	18,500	0.5652	2.3495	2.7689	0.8545	1.9427	2.94786	0.9547	1.14747	1.69787
	400	74,000	1.7888	2.2898	4.5665	0.8577	1.7621	3.5597	0.55287	0.6889	1.7999

**Table 8:** Average candidate *s* − *t* QSS paths for the SESE algorithm for 1 *Mb*.

*r*	*n*	*m*	Average Candidate s − t QSS Paths for 10 Runs								
			*P_u_* = 10			*P_u_* = 100			*P_u_* = 1000		
			*t_s_* = 100	*t_s_* = 105	*t_s_* = 110	*t_s_* = 100	*t_s_* = 105	*t_s_* = 110	*t_s_* = 100	*t_s_* = 105	*t_s_* = 110
10	100	4600	5.6	8.7	8.7	9.3	9.7	9.8	8.5	9.4	9.6
	200	18,500	7.5	9	9.5	9.5	9.5	9.5	8.5	9.5	9
	400	74,000	10	10	10	9	10	10	10	10	10
100	100	4600	37.2	53.2	53.4	58.6	62.6	58.8	59	60.2	64
	200	18,500	50.2	51.5	59.2	59.1	66.2	67.5	57.1	62	59.9
	400	74,000	55	56.2	59.8	59	64.1	62.9	60.1	62.8	63-
1000	100	4600	53	82.4	82.6	88	94.8	96.4	84	95	96.6
	200	18,500	83.3	88	94.4	92.1	96.2	97	96	97.6	96.9
	400	74,000	89.1	90.2	92.1	92.5	94.1	95.3	94.2	95.7	98.3

**Table 9:** Mean hop counts for the effective path for the SESE algorithm for 1 *Mb*.

*r*	*n*	*m*	Mean Hop Counts fo effective Path for 10 Runs								
			*P_u_* = 10			*P_u_* = 100			*P_u_* = 1000		
			*t_s_* = 100	*t_s_* = 105	*t_s_* = 110	*t_s_* = 100	*t_s_* = 105	*t_s_* = 110	*t_s_* = 100	*t_s_* = 105	*t_s_* = 110
10	100	4600	2.7	2.7	2.6	2.5	3.1	2.9	2.4	2.6	2.4
	200	18,500	1.5	2	2.5	4	2.5	2.5	3.5	3	3
	400	74,000	4.2	2.9	3.1	4.1	2.5	2.5	3	2.9	2.9
100	100	4600	2.2	3	3	3	2.8	2.4	2.8	2.8	2.8
	200	18,500	3	2.8	2.8	2.8	2.5	2.5	3	3.1	2.2
	400	74,000	5.1	3.9	3.5	4	3.1	2.9	5.2	4.7	2.2
1000	100	4600	2.8	2.8	2.6	2.6	3	2.4	2.2	2.4	3.2
	200	18,500	3.9	3	2.9	3.5	.3.1	2.8	3.2	2.9	2.9
	400	74,000	2.8	2.6	2.1	2.5	2.1	2.1	3.1	3.1	2.5

**Table 10:** Mean energy efficiency for the effective path for the SESE algorithm for 1 *Mb*.

*r*	*n*	*m*	Mean Energy Efficiency for the effective Path for 10 Runs								
			*P_u_* = 10			*P_u_* = 100			*P_u_* = 1000		
			*t_s_* = 100	*t_s_* = 105	*t_s_* = 110	*t_s_* = 100	*t_s_* = 105	*t_s_* = 110	*t_s_* = 100	*t_s_* = 105	*t_s_* = 110
10	100	4600	0.55833	0.62422	0.79478	0.755	0.66798	0.92826	0.3184	0.59678	2.21058
	200	18,500	0.7212	1.8224	5.28875	0.297	2.7963	3.26115	1.1895	3.3084	3.38985
	400	74,000	0.6322	2.7651	3.4958	3.619	1.9048	1.6107	1.2171	1.134	2.4272
100	100	4600	0.22642	0.61884	0.98046	0.56456	1.23576	1.30976	0.6487	0.70338	0.71424
	200	18,500	0.2493	1.8543	7.4257	0.431	0.7401	1.3021	0.4928	0.6337	2.0408
	400	74,000	0.2774	1.4341	2.4725	1.2175	2.7778	4.2181	1.1470	1.8654	2.1414
1000	100	4600	0.11308	0.29756	0.71988	0.45132	2.91246	0.79842	0.2263	1.16714	1.21476
	200	18,500	0.9509	1.3049	2.5281	1.4715	1.385	2.3265	0.5723	1.1746	1.6802
	400	74,000	1.0201	1.2051	2.0142	1.1542	1.8841	2.1045	1.8874	2.0149	2.9879

**Table 11:** Average candidate *s* − *t* QSS paths for the SESE algorithm for 10 *Mb*.

*r*	*n*	*m*	Average Candidate s − t QSS Paths for 10 Runs								
			*P_u_* = 10			*P_u_* = 100			*P_u_* = 1000		
			*t_s_* = 100	*t_s_* = 105	*t_s_* = 110	*t_s_* = 100	*t_s_* = 105	*t_s_* = 110	*t_s_* = 100	*t_s_* = 105	*t_s_* = 110
10	100	4600	3.9	7.1	7.1	7.3	8.1	8.2	6.9	7.8	7.9
	200	18,500	5.9	7.2	7.9	7.9	7.5	8.4	6.9	7.9	8.2
	400	74,000	7.2	7.2	7.2	7.5	8.8	8.8	8.8	8.8	8.8
100	100	4600	35.5	52.5	55.6	56.9	60.9	56.9	57.5	58.7	62.8
	200	18,500	48.5	49.9	57.5	57.6	64.6	65.9	55.5	60	57.5
	400	74,000	53.5	54.5	58.1	57.2	62.9	61.2	58.8	61.1	62
1000	100	4600	51.2	80.6	80.9	86.2	92.9	94.6	82.2	93.2	94.6
	200	18,500	81.5	86.2	92.8	90.5	94.4	95.2	94.2	95.8	95.1
	400	74,000	87.6	88.5	90.5	90.7	92.3	93.5	92.4	93.9	96.6

**Table 12:** Mean hop counts for the effective path for the SESE algorithm for 10 *Mb*.

*r*	*n*	*m*	Mean Hop Counts for the Optimal Path for 10 Runs								
			*P_u_* = 10			*P_u_* = 100			*P_u_* = 1000		
			*t_s_* = 100	*t_s_* = 105	*t_s_* = 110	*t_s_* = 100	*t_s_* = 105	*t_s_* = 110	*t_s_* = 100	*t_s_* = 105	*t_s_* = 110
10	100	4600	1.8	1.8	1.7	1.6	2.2	2	1.5	1.7	1.5
	200	18,500	1.1	1.6	2.1	3.6	2.1	2.1	3.1	2.6	2.6
	400	74,000	3.3	2.1	2.2	3.2	1.6	1.6	2.1	2.2	2.9
100	100	4600	1.3	2.1	2.1	2.1	1.9	1.5	1.9	1.9	1.9
	200	18,500	2.1	1.9	1.9	1.9	1.6	1.6	2.1	2.2	1.3
	400	74,000	4.2	4.1	2.6	3.1	2.2	2	4.3	3.8	1.3
1000	100	4600	1.9	1.9	1.7	1.7	2.1	1.5	1.3	1.5	2.3
	200	18,500	2.9	2.1	2	2.6	1.3	1.9	2.3	1	2
	400	74,000	1.9	1.7	1.2	1.6	1.2	1.2	2.2	2.2	1.6

**Table 13:** Mean energy efficiency for the effective path for the SESE algorithm for 10 *Mb*.

*r*	*n*	*m*	Mean Energy Efficiency for the Optimal Path for 10 Runs								
			*P_u_* = 10			*P_u_* = 100			*P_u_* = 1000		
			*t_s_* = 100	*t_s_* = 105	*t_s_* = 110	*t_s_* = 100	*t_s_* = 105	*t_s_* = 110	*t_s_* = 100	*t_s_* = 105	*t_s_* = 110
10	100	4600	0.33833	0.42487	0.64858	0.5895	0.4558	0.72822	0.2895	0.48657	2.0056
	200	18,500	0.5215	1.5228	5.08865	0.098	2.5967	3.19118	1.0995	3.1587	3.24988
	400	74,000	0.5424	2.4557	3.3857	3.4194	1.6048	1.3109	1.0146	1.039	2.0277
100	100	4600	0.05644	0.45887	0.68085	0.3647	1.0257	1.15977	0.4188	0.45338	0.51429
	200	18,500	0.0497	1.5547	7.1258	0.132	0.4402	1.0025	0.2929	0.6339	2.0008
	400	74,000	0.0275	1.224	2.2225	1.0075	2.5278	4.0081	1.0070	1.6157	2.0019
1000	100	4600	0.0038	0.05752	0.50987	0.20135	2.71245	0.56846	0.0264	0.9714	1.00474
	200	18,500	0.7008	1.0546	2.2285	1.2516	1.084	2.0261	0.2724	1.00464	1.3001
	400	74,000	1.0002	1.0152	1.8541	0.9541	1.6542	1.6541	1.5575	1.8545	2.6576
